# Undifferenced Kinematic Precise Orbit Determination of Swarm and GRACE-FO Satellites from GNSS Observations

**DOI:** 10.3390/s22031071

**Published:** 2022-01-29

**Authors:** Peng Luo, Shuanggen Jin, Qiqi Shi

**Affiliations:** 1 School of Communication and Information Engineering, Shanghai University, Shanghai 200444, China; Luopeng@shao.ac.cn; 2 Shanghai Astronomical Observatory, Chinese Academy of Sciences, Shanghai 200030, China; qiqishi@shao.ac.cn; 3 School of Remote Sensing and Geomatics Engineering, Nanjing University of Information Science and Technology, Nanjing 210044, China; 4 School of Surveying and Land Information Engineering, Henan Polytechnic University, Jiaozuo 454000, China; 5 School of Astronomy and Space Science, University of Chinese Academy of Sciences, Beijing 100049, China

**Keywords:** LEO satellite, kinematic precise orbit determination, GNSS, Swarm, GRACE-FO

## Abstract

Low Earth Orbit (LEO) satellites can be used for remote sensing and gravity field recovery, while precise orbit determination (POD) is vital for LEO satellite applications. However, there are some systematic errors when using the LEO satellite orbits released by different agencies in multi-satellite-based applications, e.g., Swarm and Gravity Recovery and Climate Experiment-Follow-On (GRACE-FO), as different GNSS precise orbit and clock products are used as well as processing strategies and software. In this paper, we performed undifferenced kinematic PODs for Swarm and GRACE-FO satellites simultaneously over a total of 14 days by using consistent International Global Navigation Satellite System (GNSS) Service (IGS) precise orbit and clock products. The processing strategy based on an undifferenced ionosphere-free combination and a least squares method was applied for Swarm and GRACE-FO satellites. Furthermore, the quality control for the kinematic orbits was adopted to mitigate abrupt position offsets. Moreover, the accuracy of the kinematic orbits solution was evaluated by carrier phase residual analysis and Satellite Laser Ranging (SLR) observations, as well as comparison with official orbits. The results show that the kinematic orbits solution is better than 4 cm, according to the SLR validation. With quality control, the accuracy of the kinematic orbit solution is improved by 2.49 % for the Swarm-C satellite and 6.98 % for the GRACE-D satellite when compared with their precise orbits. By analyzing the accuracy of the undifferenced kinematic orbit solution, the reliability of the LEO orbit determination is presented in terms of processing strategies and quality control procedures.

## 1. Introduction

Low Earth Orbit (LEO) satellite is a valuable platform for Earth observation due to the low altitude, short period, and rapid connection with the ground [1]. The observation data collected by the LEO satellite have been widely used in geosciences with a wide area coverage, such as crustal deformation [2,3], ocean altimetry [4], Earth gravity field recovery [5,6], space weather [7,8] and remote sensing [9,10]. To ensure successful scientific applications undertaken by the LEO satellite, the precise orbit determination (POD) for the LEO satellite is essential. With the launch of the project Topex/Poseidon, developed jointly by the National Aeronautics and Space Administration (NASA) and the Centre National d’Etudes Spatiales (CNES) in 1992, satellite-borne GPS technology has been utilized to obtain a high-accuracy centimeter-level orbit. The on-board GNSS receivers have been applied to achieve high-accuracy orbital information in the subsequent LEO satellite missions.

Kinematic POD (KPOD) of LEO satellites does not rely on any orbital dynamics model and can achieve high-precision results. Many scholars conducted high-precision KPOD. For instance, Svehla et al. [11] applied undifferenced KPOD to the CHAMP satellite-borne GPS data, and the root mean squares (RMS) of the results achieved centimeter-level in all Radial, Tangent, and Normal (RTN) directions when compared to dynamic orbit. Qin [12] performed a KPOD solution based on GRACE satellite-borne GPS data of 1 day and realized a centimeter-level orbit accuracy in all RTN directions. Ren and Schön [13] proposed a KPOD solution based on the precise point positioning (PPP) technique for the Swarm satellites, and the accuracy of the solution was consistent with the precise orbit products published by the European Space Agency (ESA).

The KPOD of LEO satellites has been widely used in many fields, particularly for global gravity field recovery [14]. With the successful implementation of gravity satellite missions, such as CHAMP (CHAllenging Minisatellite Payload), GRACE (Gravity Recovery And Climate Experiment), and GOCE (Gravity field and steady-state Ocean Circulation Explorer), the recovery in the Earth’s gravity field based on precise kinematic orbits of LEO satellites has become a major method. Weigelt et al. [15] inverted the 60th-order global time-varying gravity field by using the satellite-borne data of CHAMP. Jäggi et al. [16] recovered the global gravity field with the 18 months’ precision kinematic orbit of Swarm satellites, which could be able to fill the data gap between GRACE and GRACE-FO (GRACE Follow On). Chen et al. [17] calculated the Tongji-Grace 2018 monthly gravity field model based on the GRACE’s precise kinematic orbit by using the optimized short-arc method. Wan et al. [18] discuss the possibility of time-variable gravity field detection by using GOCE gravity gradient observations, indicating the potential of the superconducting gravity gradiometer in time-variable gravity detection.

In recent years, multi-satellite joint applications have gradually become a main trend due to the advantage of LEO satellites in many aspects. For example, due to the influence of the inclination of the satellite orbit, the re-entry period of the satellite, and the lack of on-board data, the single-satellite-based time-varying gravity field is limited in terms of accuracy, spatial resolution, and temporal resolution [19]. However, this situation can be improved by the multi-satellite observations, which can increase the coverage and density of satellite data. Additionally, when the data from one of the satellites is missing, the data from the other satellites can be well supplemented. However, most of the precise LEO satellite orbits are processed and released by different agencies with different GNSS precise orbit and clock products, which will cause systematic errors in multi-satellite-based applications. The effect factors of the orbit accuracy of LEO satellites include not only the quality of GNSS observations, but also the quality of the above-mentioned GNSS products and the processing methods of GNSS data. That is why we cannot directly use the precise kinematic orbit of GRACE-FO and Swarm satellites for the joint multi-satellite inversion of time-varying gravity fields, even the published orbits from the Deutsche GeoForschungsZentrum (GFZ) and the European Space Agency (ESA) with centimeter-level accuracy. Thus, the research on KPOD and the assessment of the orbit accuracy for multi-LEO satellites should be carried out.

This paper performs and presents a KPOD solution based on an undifferenced ionosphere-free (IF) combination for Swarm and GRACE-FO satellites simultaneously over 14 days from 1 June to 14 June 2021. The most precise GNSS precise orbit and clock products from International GNSS Service (IGS) are used for Swarm and GRACE-FO satellites as well as the same processing strategy and least squares batch processing method. To minimize the effects of abrupt position offsets that occurred in the kinematic orbit, a Reduced Dynamic POD (RDPOD) was performed along with the KPOD to facilitate subsequent quality control of the solved kinematic orbit. Finally, we demonstrated the reliability of the quality control method and the accuracy of the solved orbits by evaluating the carrier phase residual, Satellite Laser Ranging (SLR) validation and comparison with a precise ephemeris.

## 2. Methods and Data

### 2.1. Satellite-Based GNSS Observation Model

Both Swarm and GRACE-FO satellites are equipped with on-board dual-frequency GNSS receivers [20]. The pseudorange and carrier phase observations for LEO satellites relative to satellite *S* (one of GNSS satellites) at a frequency of i can be expressed as [21]:

(1) $$P_{LEO,i}^{S}=\rho_{LEO}^{S}+c\cdot \delta t_{LEO}-c\cdot \delta t^{S}+\delta \rho_{ion,i}+\delta \rho_{rel}+\delta \rho_{pco}+\delta \rho_{pco}^{S}+M_{Pi}+\varepsilon_{Pi}$$

(2) $$L_{LEO,i}^{S}=\rho_{LEO}^{S}+c\cdot \delta t_{LEO}-c\cdot \delta t^{S}+\delta \rho_{ion,i}+\delta \rho_{rel}+\delta \rho_{pco}+\delta \rho_{pco}^{S}-\lambda_{i}\cdot N_{i}+M_{Li}+\varepsilon_{Li}$$

where $P_{LEO,i}^{S}$ and $L_{LEO,i}^{S}$ are the pseudorange and carrier phase observations of the LEO satellites, respectively; $\rho_{LEO}^{S}$ is the geometric distance from the LEO satellite to the satellite *S*; c denotes the speed of light; $\delta t_{LEO}$ is the on-board receiver clock offset; $\delta t^{S}$ is the clock offset of satellite *S*; $\delta \rho_{ion,i}$ is the ionospheric delay; $\delta \rho_{rel}$ is the relativity correction; $\delta \rho_{pco}$ and $\delta \rho_{pco}^{S}$ are the antenna Phase Center Offset (PCO) of the LEO satellite and satellite *S*, respectively; $M_{Pi}$ and $M_{Li}$ are the pseudo-range and carrier phase multi-path correction, respectively; $\lambda_{i}$ is the wavelength; $N_{i}$ is ambiguity (cycle); and $\varepsilon_{Pi}$ and $\varepsilon_{Li}$ are the noises of the pseudorange and carrier phase observation, respectively.

The ionospheric delay is one of main errors for GNSS positioning and orbit determination [22,23]. To eliminate the effect of ionospheric delay, a typical ionosphere-free (IF) combination for dual-frequency pseudorange and carrier phase observation can be derived [24]:

(3) $$P_{3}=\frac{f_{1}^{2}}{f_{1}^{2}-f_{2}^{2}}\cdot P_{LEO,1}^{S}-\frac{f_{2}^{2}}{f_{1}^{2}-f_{2}^{2}}\cdot P_{LEO,2}^{S}$$

(4) $$L_{3}=\frac{f_{1}^{2}}{f_{1}^{2}-f_{2}^{2}}\cdot L_{LEO,1}^{S}-\frac{f_{2}^{2}}{f_{1}^{2}-f_{2}^{2}}\cdot L_{LEO,2}^{S}$$

where $f_{i}$ represents the frequency *i*; $P_{3}$ and $L_{3}$ are the pseudorange and carrier phase IF observation. If we combine the equations from (1) to (4), we can obtain the IF observation equation as:

(5) $$P_{3}=\rho_{LEO}^{S}+c\cdot \delta t_{LEO}-c\cdot \delta t^{S}+\delta \rho_{rel}+\delta \rho_{pco}+\delta \rho_{pco}^{S}+\frac{f_{1}^{2}\cdot M_{P1}-f_{1}^{2}\cdot M_{P2}}{f_{1}^{2}-f_{2}^{2}}+\varepsilon_{P}$$

(6) $$L_{3}=\rho_{LEO}^{S}+c\cdot \delta t_{LEO}-c\cdot \delta t^{S}+\delta \rho_{rel}+\delta \rho_{pco}+\delta \rho_{pco}^{S}+\frac{f_{1}^{2}\cdot M_{L1}-f_{1}^{2}\cdot M_{L2}}{f_{1}^{2}-f_{2}^{2}}-\lambda_{3}\cdot N_{3}+\varepsilon_{L}$$

where $N_{3}$ is ambiguity of the IF observation equation. The least squares batch processing method is commonly used in LEO satellites’ POD, which can achieve high accuracy and stability of the solution. For example, Zhao et al. [25] used the least squares batch processing method to achieve centimeter-level POD of GPS and CHAMP satellites based on PANDA (Position And Navigation Data Analysis) software developed independently by Wuhan University. Ijssel et al. [26] applied GNSS High-precision Orbit determination Software Tools (GHOST) to estimate the kinematic orbits of Swarm satellites with undifferenced observations and standard Bayesian weighted least squares estimation and realized a centimeter-level accuracy.

In this paper, a least squares batch processing method based on an undifferenced IF combination and quality control are adopted when simultaneously performing KPOD and RDPOD for Swarm and GRACE-FO satellites.

### 2.2. Data Sources and Solving Strategies

In this paper, the dual-frequency GPS observation files of Swarm (Swarm-A/B/C) and GRACE-FO satellites (GRACE-C and GRACE-D) during the day of year (DOY) 152–165 (from 1 June to 14 June) in 2021 were adopted, which were provided by the ESA and the GFZ. In the processing, the GPS precise orbit products with a 15 min sampling rate and the precise GPS clock offset products with a 30 s sampling rate were provided by IGS, and the Earth Orientation Parameter (EOP) data were provided by the Center for Orbit Determination in Europe (CODE).

The RDPODs of Swarm and GRACE-FO satellites were simultaneously carried out with KPOD, while the result of RDPOD was used as a reference to control the quality of the KPOD result. We used Bernese GNSS Software to process the data, replacing an explicit modeling of non-gravitational forces by dedicated empirical orbit parametrizations [27], as the main purpose is KPOD in this paper. The force models, data sets and processing strategies of POD are summarized in Table 1.

### 2.3. Quality Control for LEO Satellites’ Kinematic Orbit

**Figure 1:**
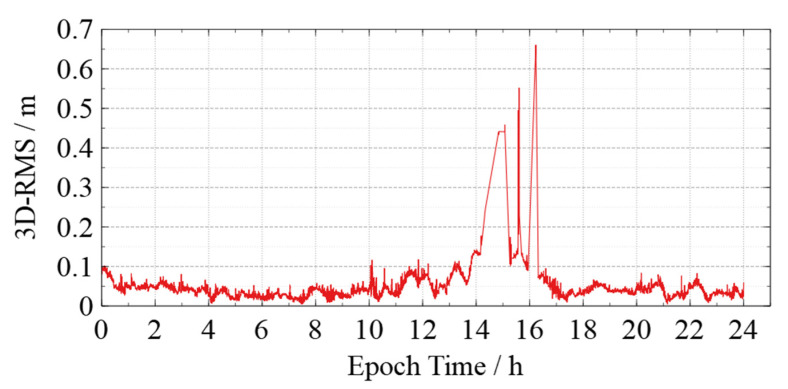
The 3-D RMS time series and jumping phenomenon in the kinematic orbit of Swarm-C occurred between 14:00 and 16:00 on DOY 162, 2021.

KPOD does not rely on any orbit dynamics model, and the accuracy of the results is strongly influenced by the quality of the GNSS observations. However, some abrupt position offsets (termed “jumping phenomenon” for simplicity hereafter) can occur at certain moments. For example, by comparing the kinematic orbit of Swarm-C solved in this paper (DOY 162, 2021) with the precise orbit published by the ESA, the 3D-RMS is obtained, as shown in Figure 1. There is a significant jumping phenomenon between 14:00 and 16:00 with reaching decimeter-level. Therefore, quality control for the kinematic orbit is necessary.

The quality control of GNSS solutions is usually carried out in the data preprocessing. For instance, Peter et al. [31] performed a KPOD solution and realized a dm-level orbit accuracy by setting proper data screening options. Ren and Schön [13] contributed an approach for the detection and repair of cycle slips in data preprocessing, which obtained good results in Swarm orbit determination. However, none of these methods can completely eliminate the jumping phenomenon.

In this paper, we used a simple method to control the quality of the final kinematic orbit. The RDPOD was simultaneously carried out with KPOD so that they could be compared to obtain an indicator of the orbit quality [32]. Additionally, the dynamic orbit was more accurate and smoother than the kinematic orbit, as the orbital dynamics were taken into account in the calculation. Therefore, we can make full use of the dynamic orbit and control the kinematic orbit’s quality with following approaches:

1. Taking the dynamic orbit as a reference, the orbital residuals are calculated by through subtracting the kinematic orbit and the dynamic orbit;
2. Filtering the kinematic orbit based on the 3σ principle, supposing the data as Gross Errors and rejecting them when the orbital residual is greater than 3σ;
3. Using Chebyshev polynomials to replace the gaps in the kinematic orbit data. However, we only fill the gaps for less than three consecutive vacancies for reducing the effect of unnecessary fitting errors.

## 3. Results and Analysis

In this paper, the performance of kinematic orbits and reduced dynamic orbits solution is evaluated by carrier phase residual analysis, SLR validation, and comparison with a precise ephemeris.

### 3.1. Carrier Phase Residual Analysis

**Figure 2:**
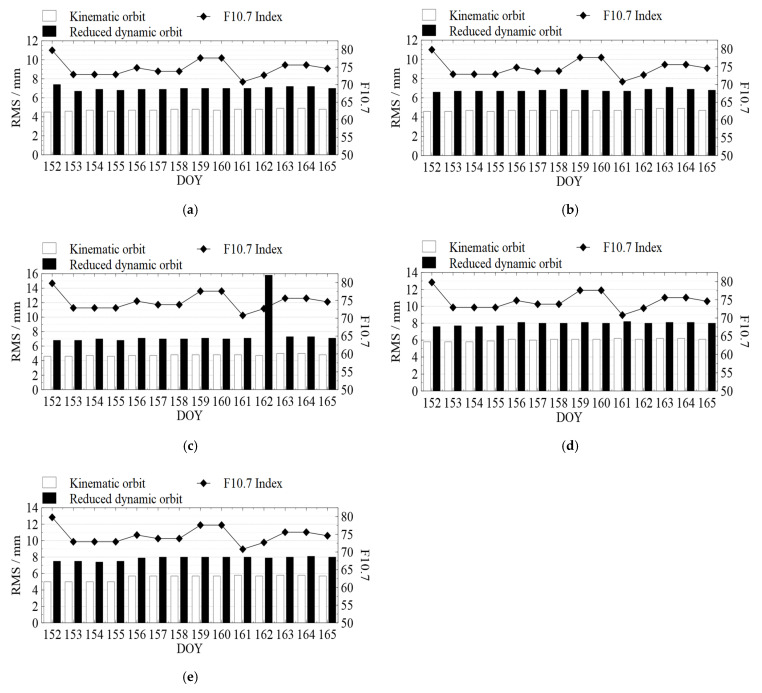
RMS of the phase observation residuals: (**a**) Swarm-A; (**b**) Swarm-B; (**c**) Swarm-C; (**d**) GRACE-C; and (**e**) GRACE-D, where white blocks and black blocks in (**a**–**e**) mean the RMS of the kinematic orbit and reduced dynamic orbit on each day, respectively. The diamond-shaped lines show the solar flux index F10.7 for each day (unit: 10^−22^ W/m^2^/Hz).

The residuals of the phase observations were calculated to evaluate the accuracy of the LEO satellite orbiting results. They were close to the observation noise level when the quality of the observations and the preprocessing results were satisfactory [33]. Figure 2 shows the residual RMS of the phase observations of Swarm and GRACE-FO satellites and the solar flux index F10.7 on that day. Meanwhile, all satellites’ mean RMS of the residuals for all phase observations are summarized in Table 2.

According to Figure 2 and Table 2, the solar flux index F10.7 fluctuated between 70 and 80 during DOY 152 to 165 in 2021, which means that no excessively intense solar activity occurred during this time. The RMS of residuals of the phase observation for Swarm satellites KPOD solution is distributed between 4 and 5 mm, while the RMS for the RDPOD solution is mostly distributed between 6.5 and 7.5 mm (except for Swarm-C with an RMS of 15.8 mm on DOY 162). The RMS of the phase observations of GRACE-FO satellites for the KPOD solution is in the range of 5.0–6.5 mm, and the RMS for the RDPOD solution is in the range of 7.5–8.5 mm.

**Figure 3:**
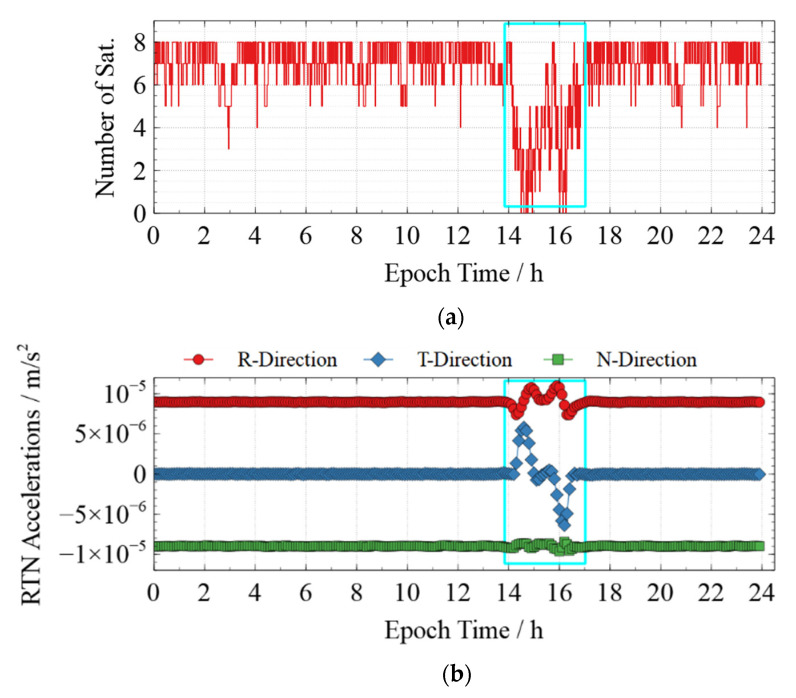
The number of observations and RTN accelerations in RDPOD for Swarm-C on DOY 162, 2021. (**a**) The number of tracked GPS satellites after data preprocessing for Swarm-C; (**b**) Estimated RTN accelerations of Swarm-C.

The phase observation residuals’ RMS for the RDPOD solution of Swarm-C on DOY 162 reached 15.8 mm, which was higher than that in any other days. Analyzing the number of tracked GPS satellites after data preprocessing and the RTN accelerations estimated by RDPOD for Swarm-C on DOY 162 (shown in Figure 3), we found that lots of observations between 14:00 and 16:00 were deleted during data preprocessing, resulting in a fewer number of observations than six in many epochs. As we set the minimum number of observations when processing data, the estimation of Epoch Clock Offsets and RTN accelerations in RDPOD are affected when the number of observations is less than six, which reduces the accuracy of RDPOD. The accuracy of KPOD is also reduced for the same reason.

Therefore, the models, data sets, and solving strategies adopted in this paper are reasonable, and the results are stable. Furthermore, the number of tracked GPS satellites in many epochs between 14:00 and 16:00 is less than five, which reduces the accuracy of RDPOD. The residual RMS of the phase observation of Swarm-C for the RDPOD solution is 15.8 mm on DOY 162, 2021.

### 3.2. Satellite Laser Ranging Validation

Each Swarm and GRACE-FO satellite is equipped with a Laser Retro-Reflector, which consists of four corner cubes mounted in a small pyramid on the underside of the spacecraft [34]. SLR is a precise technique for ranging and orbit determination [6], which can be used for external calibration and validation of the LEO satellite orbit determination with on-board GPS receiver.

The SLR technique for LEO satellites equipped with Retro-Reflectors is highly precise and has unambiguous distance measurements between the SLR station and the satellite. It is fully independent of the GNSS POD technique, so it can be used to validate the orbit of the LEO satellite by directly comparing the SLR range with the geometric distance between the SLR station and the LEO satellite’s microwave-calculated orbit [35,36].

The path of the laser is from SLR station telescopes to the Retro-Reflector mounted on the satellite and then reflects to the detector of the station. The SLR observes the time-of-flight of the laser. We will obtain a two-way range by multiplying the travel time with the speed of light [37]. The SLR range model can be described as follows [38]:

(7) $$\rho =\rho_{0}+\Delta \rho_{T}+\Delta \rho_{F}+\Delta \rho_{R}+\Delta \rho_{M}+\Delta \rho_{O}+\varepsilon$$

where ρ is the SLR range; $\rho_{0}$ is the geometric distance between the SLR station and the LEO satellite’s microwave- computed orbit; $\Delta \rho_{T}$ is the total correction caused by solid tides, pole tides, sea tides, and atmospheric tides; $\Delta \rho_{F}$ is the tropospheric range correction; and $\Delta \rho_{R}$ is the relativistic correction; and ε is the observation noise. The eccentric correction of laser reflector $\Delta \rho_{M}$ and the correction due to the station eccentricity $\Delta \rho_{O}$ are also taken into account for the range delay.

In this paper, we collected 14 days’ SLR observations of Swarm and GRACE-FO satellites stored as Normal Point (NPT) data, which were provided by the International Laser Ranging Service (ILRS). In order to ensure the quality of data processing and the reliability of the validation, the following data processing strategies (Table 3) were adopted:

1. To reduce the effect of tropospheric refraction on SLR observations, the SLR observation is excluded with satellite altitude angles less than 25°;
2. The data from the station Svetloe (1888 12350S002) and the station Irkutsk (1891 12313S007) are deducted to ensure the quality of the observations;
3. The residuals greater than 3σ during the validation based on the 3σ principle are rejected.

**Figure 4:**
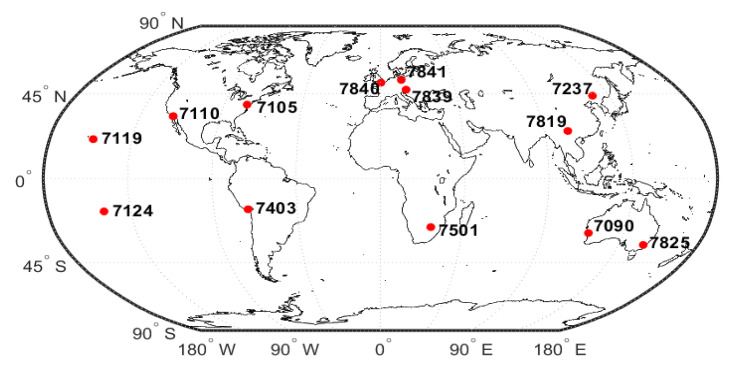
The distribution of the selected 13 SLR stations.

**Figure 5:**
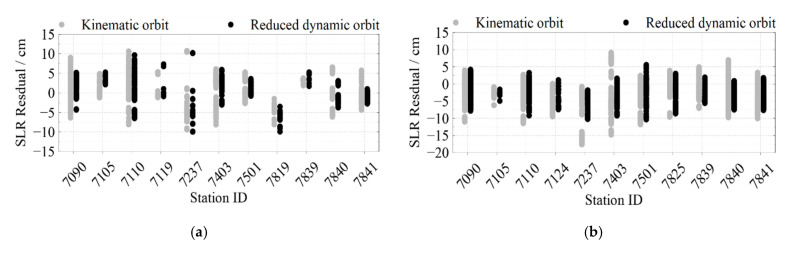

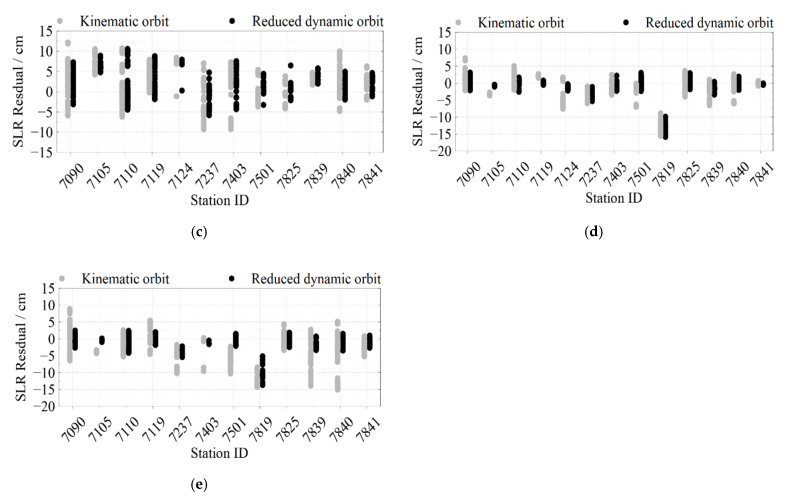
SLR residuals for Swarm and GRACE-FO satellites’ kinematic and reduced dynamic orbits from DOY 152 to 165 in 2021 at each station. (**a**) SLR residuals for Swarm-A; (**b**) SLR residuals for Swarm-B; (**c**) SLR residuals for Swarm-C; (**d**) SLR residuals for GRACE-C; (**e**) SLR residuals for GRACE-D. Gray and black points in (**a**–**e**) are the residuals of the kinematic orbit and reduced dynamic orbit, respectively.

The NPT data from selected 13 SLR stations are used, which are shown in Figure 4. Figure 5 shows the SLR residuals for each satellite’s kinematic and reduced dynamic orbit from DOY 152 to 165 in 2021 at each station, while the residuals’ mean value and their RMS are also summarized in Table 4.

According to Figure 5 and Table 4, the undifferenced kinematic orbiting accuracy of Swarm-A is 3.39 cm, and the reduced dynamic orbiting accuracy is 2.25 cm. The accuracy of undifferenced kinematic orbits for Swarm-C is 3.33 cm, and the accuracy of reduced dynamic orbits is 2.52 cm. Compared with Swarm-A and Swarm-C, Swarm-B has a lower accuracy. The accuracy of Swarm-B undifferenced kinematic orbits is 3.71 cm, and the reduced dynamic orbiting accuracy is 2.69 cm. GRACE-FO satellites have higher orbiting accuracy than Swarm satellites. The undifferenced kinematic orbiting accuracy of GRACE-C is 1.99 cm, and the reduced dynamic orbiting accuracy is 1.06 cm, while the accuracy of undifferenced kinematic orbits for GRACE-D is 3.36 cm, and the accuracy of reduced dynamic orbiting is 1.20 cm.

Meanwhile, the accuracy of SLR validation for reduced dynamic orbits is higher than the accuracy for kinematic orbits as we need to interpolate the ephemeris of LEO satellite orbits recorded in integer seconds (for example, 2021-06-13T09:48:40) to match the non-integer second-time (for example, 2021-06-13T09:48:40.487596) points recorded in the SLR observations when performing the SLR validation. Since no orbital dynamics were taken into account in the kinematic orbits’ calculation, some jumping phenomena may occur at certain moments, and the frequency and intensity of these phenomena affect the accuracy of the interpolation and thus reduce the accuracy of the SLR validation. For example, GRACE-D has a worse accuracy of undifferenced kinematic orbits than its reduced dynamic orbits due to more jumping phenomena, and these phenomena will become more evident in the next section.

### 3.3. Comparison with a Precise Ephemeris

The precise reduced dynamic orbit (with a sampling rate of 1 s) of Swarm satellites is provided by the ESA, which was solved by GHOST with an accuracy better than 2 cm [26]. The GFZ also provides a GRACE-FO precise orbit with a sampling rate of 1 s and accuracy better than 2 cm. In this section, the reduced dynamic orbits of Swarm satellites provided by the ESA and the precise orbits of GRACE-FO satellites provided by the GFZ are used as reference orbits, which are compared with our undifferenced kinematic orbits to assess the accuracy of the results.

To visualize the jumping phenomenon in the kinematic orbit and to verify the effectiveness of the quality control measures for the kinematic orbit, the following two groups were set up in this section for the orbit comparison concerning whether or not quality control was applied:

- Group 1: Applying quality control for the solved kinematic orbits before comparing with reference orbits.
- Group 2: Comparing with reference orbits without applying quality control.

**Figure 6:**
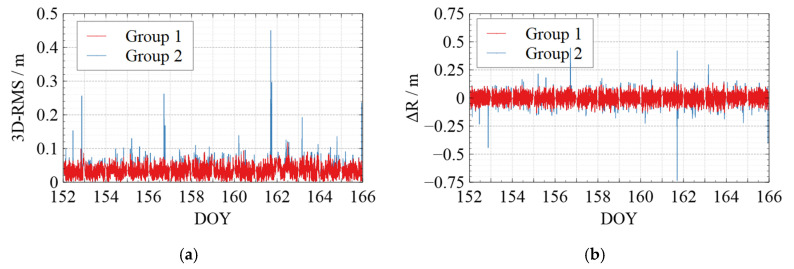

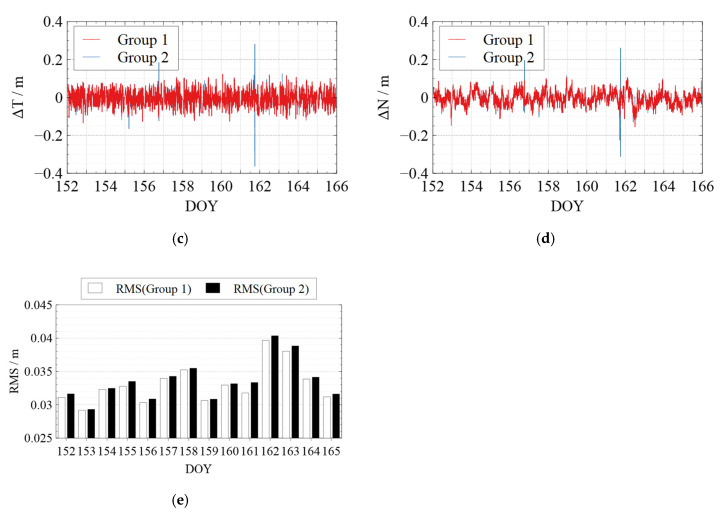
Comparison between the kinematic orbits of Swarm-A and the precise orbits provided by the ESA. (**a**) 3D-RMS of the comparison results; (**b**) Difference in Radial direction; (**c**) Difference in Tangent direction; (**d**) Difference in Normal direction; (**e**) Average 3D-RMS statistics of the comparison results. The red line shows the results of Group 1, while the blue line shows the results of Group 2 in (**a**–**d**). The white blocks and black blocks in (**e**) represent the RMS of Group 1 and Group 2 on each day, respectively.

**Figure 7:**
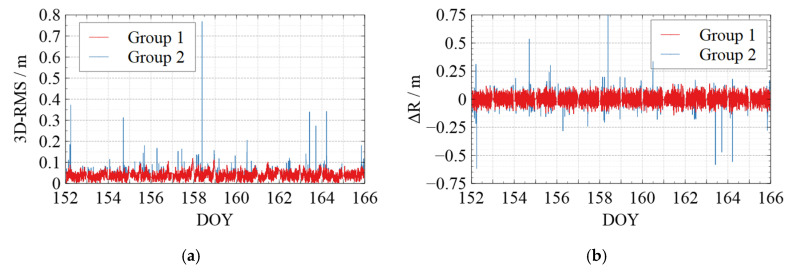

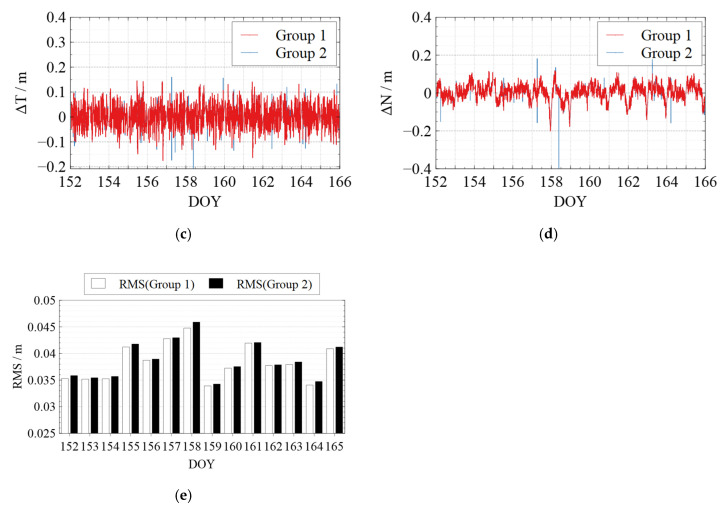
Comparison between the kinematic orbits of Swarm-B and the precise orbits provided by the ESA. (**a**) 3D-RMS of the comparison results; (**b**) Difference in Radial direction; (**c**) Difference in Tangent direction; (**d**) Difference in Normal direction; (**e**) Average 3D-RMS statistics of the comparison results. Where the red line shows the results of Group 1 while the blue line shows the results of Group 2 in (**a**–**d**). The white blocks and black blocks in (**e**) represent the RMS of Group 1 and Group 2 on each day, respectively.

**Figure 8:**
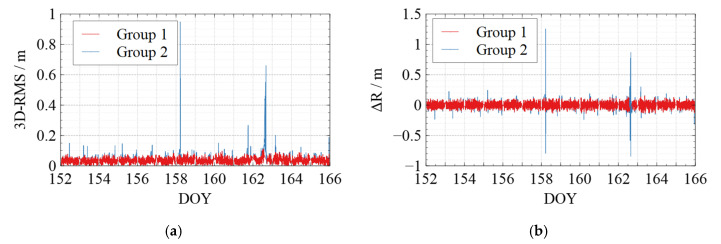

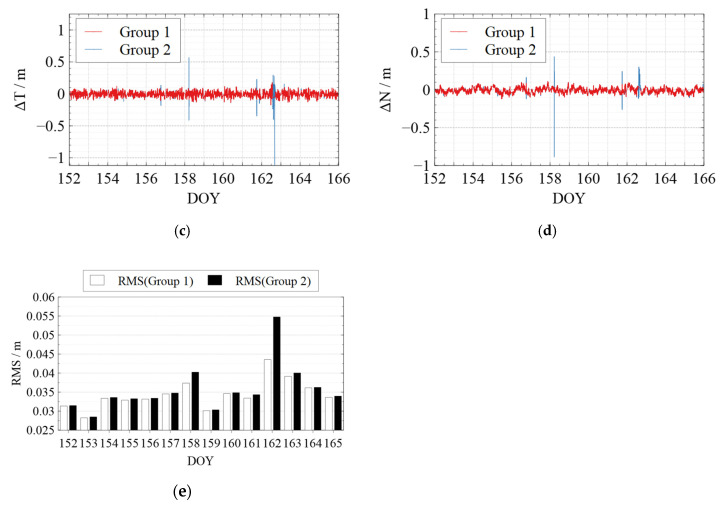
Comparison between the kinematic orbits of Swarm-C and precise orbits provided by the ESA. (**a**) 3D-RMS of the comparison results; (**b**) Difference in Radial direction; (**c**) Difference in Tangent direction; (**d**) Difference in Normal direction; (**e**) Average 3D-RMS statistics of the comparison results. The red line shows the results of Group 1, while the blue line shows the results of Group 2 in (**a**–**d**). The white blocks and black blocks in (**e**) represent the RMS of Group 1 and Group 2 on each day, respectively.

**Figure 9:**
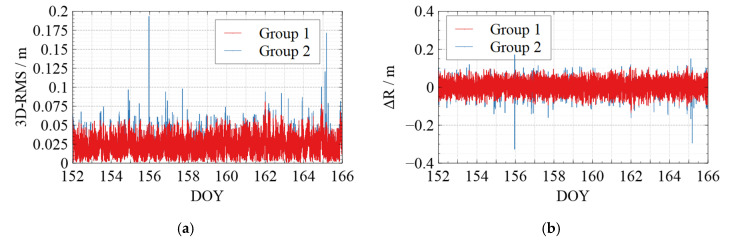

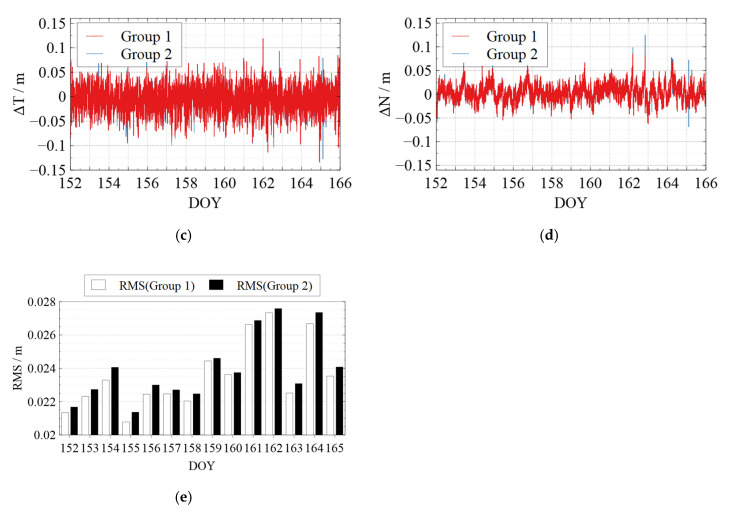
Comparison between the kinematic orbits of GRACE-C and the precise orbits provided by the GFZ. (**a**) 3D-RMS of the comparison results; (**b**) Difference in Radial direction; (**c**) Difference in Tangent direction; (**d**) Difference in Normal direction; (**e**) Average 3D-RMS statistics of the comparison results. Where the red line shows the results of Group 1 while the blue line shows the results of Group 2 in (**a**–**d**). The white blocks and black blocks in (**e**) represent the RMS of Group 1 and Group 2 on each day, respectively.

**Figure 10:**
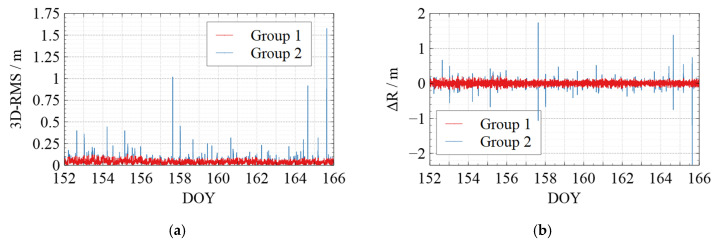

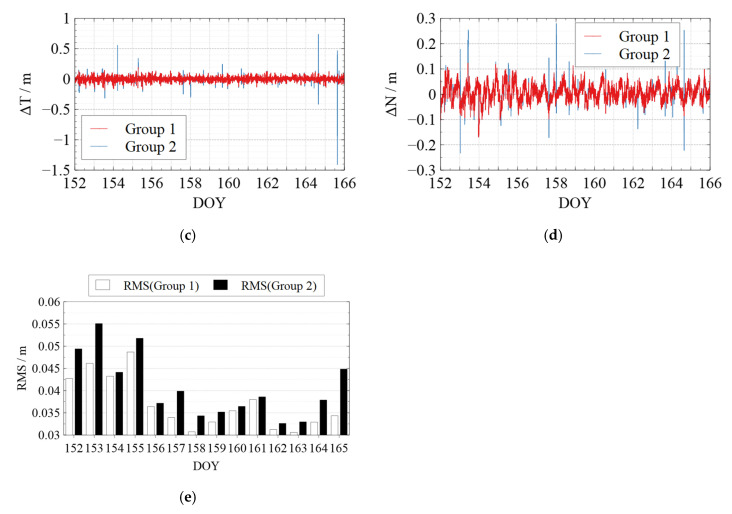
Comparison between the kinematic orbits of GRACE-D and the precise orbits provided by the GFZ. (**a**) 3D-RMS of the comparison results; (**b**) Difference in Radial direction; (**c**) Difference in Tangent direction; (**d**) Difference in Normal direction; (**e**) Average 3D-RMS statistics of the comparison results. The red line shows the results of Group 1, while the blue line shows the results of Group 2 in (**a**–**d**). The white blocks and black blocks in (**e**) represent the RMS of Group 1 and Group 2 on each day, respectively.

Figure 6, Figure 7, Figure 8, Figure 9 and Figure 10 show the comparison of the undifferenced kinematic orbits with the reference orbits for Swarm and GRACE-FO satellites. We set up colorful comparisons in these figures to highlight the RMS differences between the two groups and the orbit differences in each RTN direction. The RMS of the KPOD results for each satellite in different groups is also summarized and listed in Table 5. As we can see in Figure 6, Figure 7, Figure 8, Figure 9 and Figure 10 and Table 5, in Group 2, the RMS of the undifferenced kinematic orbits for Swarm-A/B/C are between 3.0 and 5.0 cm in RTN directions, with total 3-D RMS between 2.5 and 5.0 cm for each day. The RMS of the undifferenced kinematic orbit of GRACE-C in RTN directions ranges from 1.5 to 3.0 cm, and the total 3D-RMS are in the range of 2.0–2.8 cm for all days. The RMS of the undifferenced kinematic orbit of GRACE-D, as the leading satellite of the GRACE-FO binary system, is slightly worse with the RMS from 3.0 to 5.5 cm. Therefore, the accuracy of the kinematic orbit calculated in this paper is better than 5 cm for Swarm satellites and better than 5.5 cm for GRACE-FO satellites.

According to the comparison of Group 1 and Group 2, different levels of jumping phenomena occurred in the kinematic orbit for both Swarm and GRACE-FO satellites, some of which reached the decimeter-level, and some even reached a meter-level. However, the results of Group 1 show that the quality control method can effectively suppress the orbital jumps. The Swarm-C satellites (on DOY 162 in 2021) and GRACE-D satellites (e.g., on DOY 153, 157, 164, and 165 in 2021) with more frequent orbital jumps are taken as an example. According to Figure 8e and Figure 10e, after we took quality control measures, the total 3-D RMS of the kinematic orbit for Swarm-C on DOY 162 was decreased from 5.5 cm to 4.4 cm with about 20%. The 3D-RMSs of kinematic orbits for GRACE-D on DOY 153, 157, 164, and 165 were decreased from 5.5 cm, 4.0 cm, 3.8 cm, and 4.5 cm to 4.6 cm, 3.4 cm, 3.3 cm, and 3.4 cm, respectively with about 22 %. This shows that the quality control for kinematic orbits adopted in this paper has a significant effect.

Meanwhile, from Table 5, after quality control for the kinematic orbit, the 3D-RMS of the kinematic orbit is reduced from 3.57 cm to 3.53 cm for Swarm-A, from 4.11 cm to 4.03 cm for Swarm-B, and from 3.61 cm to 3.52 cm for Swarm-C. Therefore, the quality control method adopted in this paper improved the kinematic orbit accuracy by reducing the frequency and degree of orbital jumps, which can be seen in Figure 6, Figure 7, Figure 8, Figure 9 and Figure 10.

## 4. Conclusions

In this paper, the KPOD for Swarm and GRACE-FO satellites was carried out simultaneously for a total of 14 days from DOY 152 to 165 in 2021 with the same processing strategy based on an undifferenced IF linear combination and a least squares batch processing method. To suppress the jumping phenomenon of the kinematic orbit and improve the accuracy of the final solution, RDPOD was performed, along with KPOD, and the calculated reduced dynamic orbit was used as the benchmark for quality control of the kinematic orbit. The accuracy of the results was evaluated by three approaches: residual analysis of phase observations, SLR validation, and comparison with a precise ephemeris. The following conclusions were obtained:

1. The residuals of phase observations for Swarm and GRACE-FO satellites show that the models, data sets, and processing strategies are reasonable, and the orbit determination is stable.

2. According to the results of SLR validation, the accuracy of the kinematic orbit and the reduced dynamic orbit both achieved the centimeter-level. The accuracy of the kinematic orbit and the reduced dynamic orbit of Swarm satellites is better than 4 cm. The accuracy of the kinematic orbit of GRACE-FO satellites is better than 4 cm, and the accuracy of their reduced dynamic orbit is better than 2 cm.

3. After quality control for the kinematic orbit, the jumping phenomenon in the kinematic orbit was alleviated. By comparing with the precise ephemeris, the kinematic orbit accuracy was improved by 2.49% for Swarm-C and 6.98 for GRACE-D after the quality control.

## Figures and Tables

**Table 1:** Models, data sets and processing strategies for KPOD and RDPOD.

	Model	Description
Force models	Earth gravity field	EGM2008(120 × 120) [28]
	N-body	JPL DE405
	Solid earth tides and pole tide	IERS 2010 Conversations [29]
	Ocean tides	FES2004 [30]
	Relativity	IERS 2010 Conversations [29]
Data sets	Precise GPS orbits	Provided by IGS (Sampling rate: 15 min)
	Precise GPS clock offset products	Provided by IGS (Sampling rate: 30 s)
	Earth orientation	Provided by CODE
	GPS PCO/PCV models	igs14.atx
	Weight for phase observations	cos^2^(z) ^1^
	Sampling of GPS observations	10 s
	Minimum number of observations per epoch	6
	Elevation cutoff	5°
Estimated parameters	Epoch state vector	Both KPOD and RDPOD will estimate
	Epoch clock offsets	Both KPOD and RDPOD will estimate
	Phase ambiguities	Both KPOD and RDPOD will estimate with float-solution
	Pseudo-random pulses	Only RDPOD will estimate every 15 min
	RTN empirical acceleration	Only RDPOD will estimate every 6 min

^1^ z is the zenith angle of the GPS satellite.

**Table 2:** All satellites’ average RMS of the residuals for all phase observations.

Satellite	Orbit	Average Residual RMS (mm)
Swarm-A	Kinematic	4.74
	Reduced dynamic	7.01
Swarm-B	Kinematic	4.71
	Reduced dynamic	6.78
Swarm-C	Kinematic	4.76
	Reduced dynamic	7.66
GRACE-C	Kinematic	6.04
	Reduced dynamic	7.94
GRACE-D	Kinematic	5.52
	Reduced dynamic	7.84

**Table 3:** SLR data processing strategies.

	Models/References
Station coordinates	SLRF2014
Solid earth and pole tides	IERS2010 [29]
Sea tide loading	FES2004 [30]
Tropospheric refraction	Marini-Murry models [39]
Relativity	IERS2010 [29]
Eccentric correction of Swarm	Provided by ESA
Eccentric correction of GRACE-FO	Provided by ILRS
Elevation cutoff	25°

**Table 4:** Summary for SLR residuals.

Satellite	Number of NPT	Orbit	Residuals’ Average RMS (cm)
Swarm-A	718	Kinematic	3.39
		Reduced dynamic	2.25
Swarm-B	1975	Kinematic	3.71
		Reduced dynamic	2.69
Swarm-C	752	Kinematic	3.33
		Reduced dynamic	2.52
GRACE-C	990	Kinematic	1.99
		Reduced dynamic	1.06
GRACE-D	774	Kinematic	3.36
		Reduced dynamic	1.20

**Table 5:** RMS with different groups comparing kinematic orbit and reference orbit.

Satellite	Groups	3D-RMS (cm)	R-RMS (cm)	T-RMS (cm)	N-RMS (cm)	Improvement
Swarm-A	Group 1	3.53	3.30	3.97	3.28	1.12%
	Group 2	3.57	3.39	3.99	3.29	
Swarm-B	Group 1	4.03	3.46	4.39	4.18	1.95%
	Group 2	4.11	3.60	4.48	4.20	
Swarm-C	Group 1	3.52	3.27	3.85	3.41	2.49%
	Group 2	3.61	3.42	3.92	3.45	
GRACE-C	Group 1	2.39	2.81	2.56	1.63	1.65%
	Group 2	2.43	2.90	2.58	1.64	
GRACE-D	Group 1	3.60	4.06	3.57	3.12	6.98%
	Group 2	3.87	4.56	3.74	3.19	

## Data Availability

The dual-frequency GPS observation data and precise dynamic orbit of Swarm satellites used in this paper can be downloaded from http://swarm-diss.eo.esa.int (accessed on 1 October 2021). The dual-frequency GPS observation data and precise orbit of GRACE-FO satellites used in this paper can also be downloaded from ftp://isdcftp.gfz-potsda.m.de/grace-fo/ (accessed on 1 October 2021).
